# Mutations and karyotype in myelodysplastic syndromes: *TP53* clusters with monosomal karyotype, *RUNX1* with trisomy 21, and *SF3B1* with inv(3)(q21q26.2) and del(11q)

**DOI:** 10.1038/s41408-017-0017-8

**Published:** 2017-12-18

**Authors:** Ayalew Tefferi, Dame Idossa, Terra L. Lasho, Mythri Mudireddy, Christy Finke, Sahrish Shah, Maura Nicolosi, Mrinal M. Patnaik, Animesh Pardanani, Naseema Gangat, Curt A. Hanson, Rhett P. Ketterling

**Affiliations:** 1 Division of Hematology, Departments of Internal and Laboratory Medicine, Mayo Clinic, Rochester, MN USA; 2 Division of Hematopathology, Departments of Internal and Laboratory Medicine, Mayo Clinic, Rochester, MN USA; 3 Division of Cytogenetics, Departments of Internal and Laboratory Medicine, Mayo Clinic, Rochester, MN USA

Next-generation sequencing (NGS) studies have now established the presence of sometimes multiple somatic mutations in the majority of patients with myelodysplastic syndromes (MDS)^1, 2^. Some of these mutations, including *ASXL1*, *TP53*, *RUNX1*, *EZH2*, and *SRSF2*, have been shown to adversely affect overall or leukemia-free survival, independent of each other and conventional risk models^2^. More recent studies have further suggested associations of certain mutations in MDS with specific cytogenetic abnormalities. In this regard, one study employed NGS in 22 MDS patients with der(1;7)(q10;p10) and 32 with −7/del(7q)^3^; the most frequently mutated genes in the former were *RUNX1* (41%), *ASXL1* (23%), *EZH2* (18%), and *DNMT3A* (18%) and in the latter *TP53* (28%), *ASXL1* 28%, *SETBP1* (22%), and *TET2* (19%). Accordingly, the authors suggested an association between der(1;7)(q10;p10) and *RUNX1* mutations^3^. In another NGS study of 43 patients with del(5q)-associated MDS, recurrent mutations among 22 patients with del(5q) syndrome included *ASXL1* (14%), *TET2* (14%), *SF3B1* (9%), *TP53* (5%), *RUNX1* (5%), *DNMT3A* (5%), and *WT1* (5%)^4^; more advanced cases displayed higher frequency of *TP53* mutations. Such observations carry both pathogenetic and practical relevance, especially in deciphering the prognostic interaction between mutations and karyotype.

In a recent communication, we reported on 179 MDS patients in whom information was available for NGS-derived mutational status, and showed an adverse overall and leukemia-free survival impact from *ASXL1, SETBP1*, or *TP53* mutations/variants and *SRSF2, IDH2, CSF3R* mutations/variants, respectively^2^. The prognostic contribution of these mutations was independent of co-existing mutations, number of mutations, age, and, for the large part, the revised international prognostic scoring system (IPSS-R)^2^; an apparent association between *SF3B1* mutations and favorable prognosis was no longer evident after analysis was adjusted for IPSS-R. In the current study, we sought to discover specific associations between mutations and karyotype and clarify the inter-independent prognostic contribution of mutations vs. karyotype.

The study population (*N* = 179) consisted of patients with primary MDS who were informative for both karyotype and NGS data. The diagnosis of MDS and leukemic transformation was according to the 2008 World Health Organization (WHO) criteria^5^. Cytogenetic analysis and reporting was done according to the International System for Human Cytogenetic Nomenclature (ISCN) criteria^6^. Cytogenetic reports were re-reviewed and grouped into categories that are found to be informative, on preliminary analysis of associations with specific mutations. A 27-gene panel NGS study was performed on bone marrow DNA specimens, and queried genes included *TET2, DNMT3A, IDH1, IDH2*, *ASXL1, EZH2, SUZ12*, *SRSF2, SF3B1, ZRSR2, U2AF1*, *PTPN11, TP53, SH2B3, RUNX1, CBL, NRAS, JAK2, CSF3R, FLT3, KIT, CALR, MPL*, *NPM1, CEBPA, IKZF1*, and *SETBP1*. Altered DNA sequences were deemed as mutations or variants, if they were associated with a hematologic malignancy (as identified by COSMIC database), or if they have not been associated with a dbSNP. All statistical analyses considered clinical and laboratory parameters obtained at the time of mutation screening.

Clinical and laboratory features of the 179 study patients included median age 73 years (range 28–96), males 68%, median hemoglobin 10 g/dl (range 6.9–14.8), median leukocyte count 3.6 × 10^9^/l (range 0.8–20), median platelet count 91 × 10^9^/l (range 4–599). Risk distribution according to IPSS-R was very high 11%, high 18%, intermediate 17%, low 38%, and very low 16%. Abnormal karyotype was reported in 107 (60%) patients with the most frequent being normal karyotype (*n* = 78; 43.6%), monosomal karyotype (*n* = 22; 12.3%), sole +8 (*n* = 14; 7.8%), sole del(5q) (*n* = 13; 7.3%), sole −7/del(7q) (*n* = 6; 3.4%), sole del(11q) (*n* = 5; 2.8%), sole +21 (*n* = 4; 2.2%), sole trisomies other than +8 and +21 (*n* = 5; 2.8%), sole del(20q) (*n* = 4; 2.2%), sole inv(3)(q21q26.2) (*n* = 2; 1.1%); complex non-monosomal (*n* = 3; 1.7%), der(1;7)(q10;p10) (*n* = 3; 1.7%), and other sole or double abnormalities (*n* = 20; 11.2%). At least one mutation/variant was seen in 147 (82%) patients; 58 (32%) patients harbored one, 48 (27%) two, and 41 (23%) three or more. Mutations/variants detected included *ASXL1* (*n* = 53; 30%), *TET2* (*n* = 44; 25%), *SF3B1* (*n* = 36; 20%), *U2AF1* (*n* = 28; 16%), *SRSF2* (*n* = 28; 16%), *TP53* (*n* = 23; 13%), *RUNX1* (*n* = 19; 11%), *DNMT3A* (*n* = 18; 10%), *IDH2* (*n* = 11; 6%), *EZH2* (*n* = 7; 4%), *CEBPA* (*n* = 6; 3%), *SETBP1* (*n* = 5; 3%), *IDH1* (*n* = 5; 3%), *CSF3R* (*n* = 5; 3%), *KIT* (*n* = 3; 2%), *CBL* (*n* = 2; 1%), *JAK2* (*n* = 2; 1%), *CALR* (*n* = 1; 0.5%), and *FLT3* (*n* = 1; 0.5%).

Table 1 lists mutations which showed significant associations with specific cytogenetic categories. The most notable associations were between monosomal karyotype and *TP53* mutations (*p* < 0.0001; mutational frequency of 82% vs. <10% in all other abnormal cytogenetic categories), *RUNX1* and +21 (*p* < 0.0001; mutational frequency of 100% vs. <35% in all other abnormal cytogenetic categories), and *SF3B1* and del(11q) and inv(3)(q21q26.2) (*p* = 0.0001; mutational frequency of 80% and 100%, respectively, vs. <20% in all other abnormal cytogenetic categories). Patients with trisomy 21 also frequently harbored *ASXL1* (75%) and *SRSF2* (75%) mutations. Other associations included *ASXL1* with +8, *SRSF2* with other sole trisomies, −7/del(7q) with *IDH1* and *U2AF1* mutations. The close association between *TP53* mutations and monosomal karyotype was not further modified by the presence or absence of monosomy 17, as part of their monosomal karyotype; 7 (32%) of the 22 cases with monosomal karyotype harbored monosomy 17. Similarly, the specific type of *RUNX1* mutation did not affect its association pattern with trisomy 21; among the 19 cases with *RUNX1* mutations, 13 involved the runt domain, 10 (53%) were frameshift, 7 (37%) missense, and 1 nonsense.

**Table 1:** Cytogenetic categories in 179 patients with primary myelodysplastic syndromes and significantly associated mutations

Mutations	All patients (*n* = 179)	Normal karyotype *n* = 78 (44%)	Monosomal karyotype *n* = 22 (12%)	Sole +8 *n* = 14 (8%)	Sole 5q− *n* = 13 (7%)	Sole −7/7q− *n* = 6 (3%)	Sole 11q− *n* = 5 (3%)	Sole 20q− *n* = 4 (2%)	Sole +21 *n* = 4 (2%)	Other sole trisomies *n* = 5 (3%)	Sole inv(3) *n* = 2 (1%)	Der(1;7) *n* = 3 (2%)	Complex non-monosomal *n* = 3 (2%)	Others *n* = 20 (11%)	*P*-value
*ASXL1*; *n* (%)	53 (30%)	25 (32%)	1 (5%)	7 (50%)	1 (8%)	2 (33%)	1 (20%)	1 (25%)	3 (75%)	0	0	0	1 (33%)	8 (40%)	0.02
*SF3B1*; *n* (%)	36 (20%)	24 (31%)	1 (5%)	2 (14%)	0	0	4 (80%)	0	0	0	2 (100%)	0	0	3 (15%)	0.0001
*SRSF2*; *n* (%)	28 (16%)	14 (18%)	0	2 (14%)	0	1 (17%)	1 (20%)	0	3 (75%)	3 (60%)	0	0	0	4 (20%)	0.006
*U2AF1*; *n* (%)	28 (16%)	9 (12%)	1 (5%)	3 (21%)	0	4 (67%)	0	2 (50%)	0	2 (40%)	0	0	2 (67%)	5 (25%)	0.0009
*TP53*; *n* (%)	23 (13%)	3 (4%)	18 (82%)	0	1 (8%)	0	0	0	0	0	0	0	0	1 (5%)	<0.0001
*RUNX1*; *n* (%)	19 (11%)	5 (6%)	1 (5%)	3 (21%)	0	2 (33%)	0	1 (25%)	4 (100%)	0	0	1 (33%)	0	2 (10%)	<0.0001
*IDH1*; *n* (%)	5 (3%)	2 (3%)	0	0	0	2 (33%)	0	0	0	1 (20%)	0	0	0	0	0.004

**Fig. 1:**
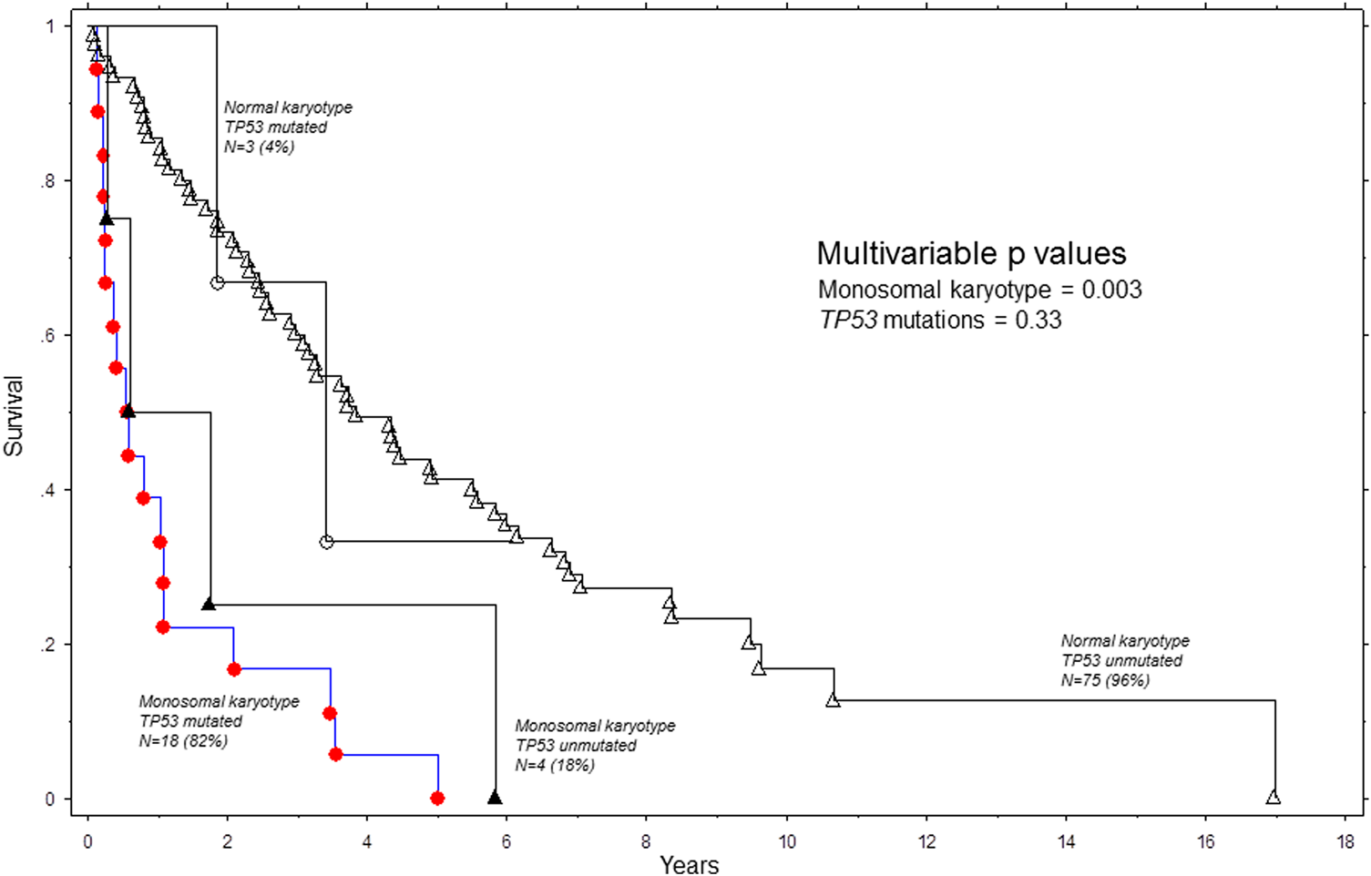
Overall survival of 100 patients with primary myelodysplastic syndromes and either normal or monosomal karyotype, stratified by the presence or absence of *TP53* mutations

Given the adequate number of informative cases with monosomal karyotype (*n* = 22), we examined its prognostic interaction with *TP53* mutations; in multivariable analysis that included these two variables, among 100 cases with either monosomal (*n* = 22) or normal (*n* = 78) karyotype, the prognostic contribution of *TP53* mutations (HR 1.4; 95% CI 0.7–3.0; *p* = 0.33) was overridden by that of monosomal karyotype (HR 2.9; 95% CI 1.4–5.9; *p* = 0.003) (Fig. 1). Similar results were obtained when the entire cohort of 179 study patients were included in the multivariable model. Furthermore, in univariate analysis, monosomal karyotype (*p* = 0.04) and not *TP53* mutations (*p* = 0.07) predicted leukemic transformation.

The observations from the current study carry both pathogenetic and prognostic relevance. The association between monosomal karyotype and *TP53* mutations has also been recognized in the setting of acute myeloid leukemia (AML)^7, 8^, and raises the possibility of mutation-induced genetic/chromosome instability as the cause of the particular cytogenetic abnormality. Furthermore, the current study suggests that the adverse impact of *TP53* mutations might be accounted for by its association with monosomal karyotype, whose independent prognostic contribution in MDS has previously been stressed^9, 10^. The association between *RUNX1* mutations and trisomy 21 was also previously reported in AML^11^, chronic myeloid leukemia,^12^ and B-cell acute lymphoblastic leukemia^13^. Our observation on the association of *SF3B1* mutations and inv(3)(q21q26.2) is novel and consistent with our previous observation of the same in chronic myelomonocytic leukemia^14^. As has also been previously noted in MDS^15^, *SF3B1* mutations were also associated with del(11q), which is prognostically different from inv(3)(q21q26.2). These observations warrant careful attention to karyotype, when asserting the prognostic impact of mutation in MDS and other myeloid malignancies.
